# Exploring Tumor-Promoting Qualities of Cancer-Associated Fibroblasts and Innovative Drug Discovery Strategies With Emphasis on Thymoquinone

**DOI:** 10.7759/cureus.53949

**Published:** 2024-02-10

**Authors:** Jabir Padathpeedika Khalid, Taniya Mary Martin, Lavanya Prathap, Milind Abhimanyu Nisargandha, Nisha Boopathy, Meenakshi Sundaram Kishore Kumar

**Affiliations:** 1 Department of Physiology, Saveetha Medical College and Hospital, Saveetha Institute of Medical and Technical Sciences, Saveetha University, Chennai, IND; 2 Department of Anatomy, Biomedical Research Unit and Laboratory Animal Centre, Saveetha Dental College and Hospital, Saveetha Institute of Medical and Technical Sciences, Saveetha University, Chennai, IND; 3 Department of Anatomy, Saveetha Medical College and Hospital, Saveetha Institute of Medical and Technical Sciences, Saveetha University, Chennai, IND; 4 Department of Community Medicine, Saveetha Medical College and Hospital, Saveetha Institute of Medical and Technical Sciences, Saveetha University, Chennai, IND

**Keywords:** fibroblast, flavonoids, thymoquinone, paracrine signaling, cancer associated fibroblast, stroma, tumour microenvironment

## Abstract

Tumor epithelial development and chemoresistance are highly promoted by the tumor microenvironment (TME), which is mostly made up of the cancer stroma. This is due to several causes. Cancer-associated fibroblasts (CAFs) stand out among them as being essential for the promotion of tumors. Understanding the fibroblastic population within a single tumor is made more challenging by the undeniable heterogeneity within it, even though particular stromal alterations are still up for debate. Numerous chemical signals released by tumors improve the connections between heterotypic fibroblasts and CAFs, promoting the spread of cancer. It becomes essential to have a thorough understanding of this complex microenvironment to effectively prevent solid tumor growth. Important new insights into the role of CAFs in the TME have been revealed by recent studies. The objective of this review is to carefully investigate the relationship between CAFs in tumors and plant secondary metabolites, with a focus on thymoquinone (TQ). The literature published between 2010 and 2023 was searched in PubMed and Google Scholar with keywords such as TQ, TME, cancer-associated fibroblasts, mechanism of action, and flavonoids. The results showed a wealth of data substantiating the activity of plant secondary metabolites, particularly TQ's involvement in blocking CAF operations. Scrutinized research also clarified the wider effect of flavonoids on pathways related to cancer. The present study highlights the complex dynamics of the TME and emphasizes the critical role of CAFs. It also examines the possible interventions provided by secondary metabolites found in plants, with TQ playing a vital role in regulating CAF function based on recent literature.

## Introduction and background

The normal physiological balance in substituting the nutrient and associated blood products is eventually maintained by the cooperation, between the normal epithelia and their active fibroblastic counterparts [1]. Thus, highly determined and integrated cell signaling controls wound healing, epithelial proliferation, and immunological manifestations by altering the stromal functions using the paracrine mechanism [2]. This regulated metabolic conversation is a requisite for the integrity and differentiation of the stromal-associated cells [3]. Meanwhile, unregulated signaling might lead to improper results, such as cancers and autoimmune diseases. The role of stromal cells in cancer proliferation stemmed from the seed-soil hypothesis that assumed the stroma as soil and the cancer cells as seeds. Hart and Fiddler suggested that the seeds (tumors) are highly influenced by soil (tissue microenvironment) and selectively exhibit higher growth on particular soils. These cancer cells strongly prefer a niche for their growth and the chemical interaction between them is the research interest for novel drug development [4]. Since these niches serve as the site of metastasis in later stages and characteristically revert the embryonic functions even at the later stage. Ultimately, the cancer cells themselves could neither achieve angiogenesis nor signaling to the rest of the nearby tissues.

In this review, we explain the molecular mechanism of thymoquinone (TQ) with the actions of flavonoids. We also substantiate the anti-oncogenic activities of these compounds by reviewing previous studies.

We searched for articles from Pubmed and Google Scholar (2010-2023) using the keywords TQ, tumor microenvironment (TME), cancer-associated fibroblasts (CAFs), mechanism of action, and flavonoids, using Boolean search. A total of 137 articles were selected for the review process based on the keywords.

**Figure 1:**
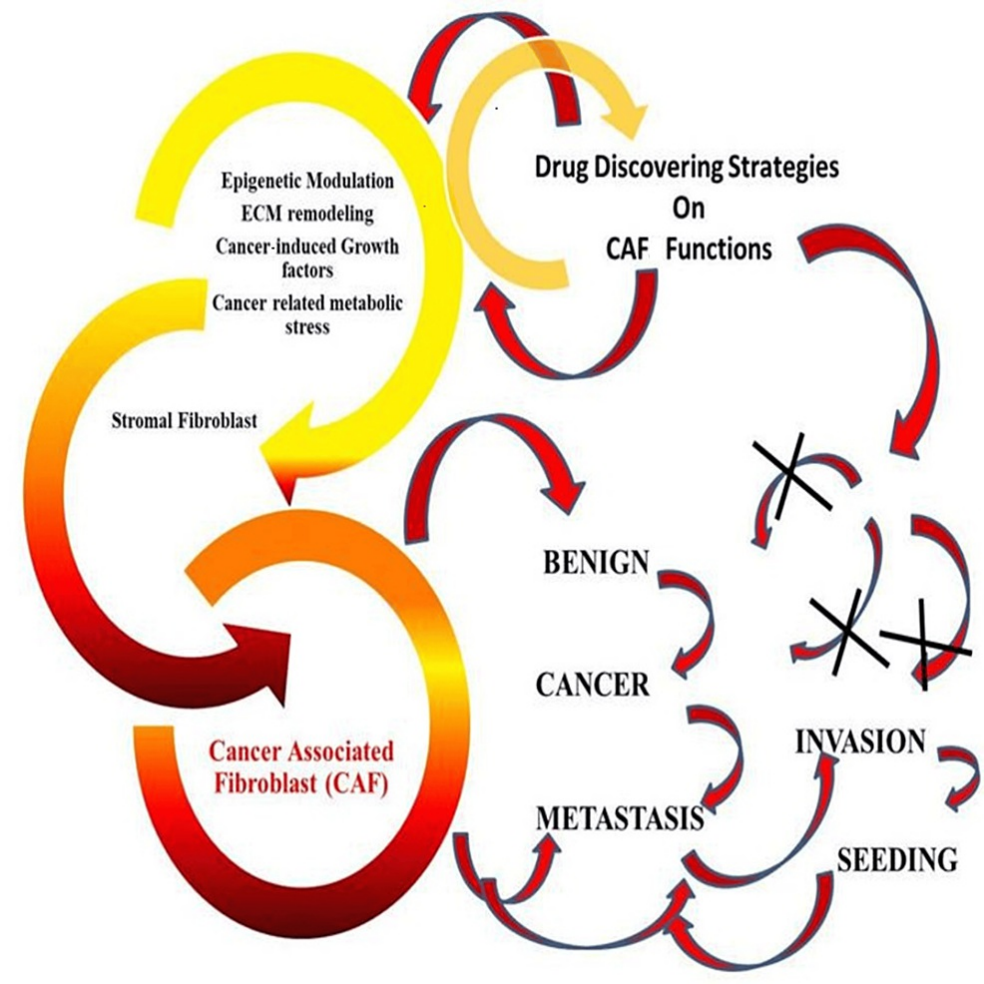
Dual nature of fibroblasts in tumors. Fibroblasts present in stroma around the cancer cells are highly regulated by them. Tumors modify their functions for nurturing the CAF. Meanwhile, CAF offers excellent tools for orchestrating drug-discovery strategies against cancers. CAF: Cancer-associated fibroblast; ECM: Extracellular matrix Image Credit: Meenakshi Sundaram

Significance of CAFs in TME

The signal rewiring in the cancer microenvironment is strongly dependent on the non-cancerous stromal cells, and recent literature has shown its molecular mechanism extensively [5]. The CAFs are one of the subsets of the TME and are derived from different sources such as endothelium, epithelium, or pre-existing fibroblasts [6]. The mitochondria of tumor cells and the changed catabolism of that fibroblast together influence tumor growth positively [7]. By the tricarboxylic (TCA) cycle, on oxidative phosphorylation (OXPHOS), catabolic fibroblasts give the essential fuels (L-lactate, ketones, glutamine) to anabolic cancer cells. Hence, the altered glycolytic reprogramming governs the fate of oncogene activation and inflammation at TME [8]. Oncogenes cause the formation of CAF phenotype from other fibroblasts through oxygen metabolism [9]. Caveolin-1 (Cav-1) loss and an increase in MCT4 genes of neighboring fibroblasts that activate tumor-like metabolism in stromal fibroblasts [10]. Similar to other carcinoma, the CAFs also showed functional similarity based on rat sarcoma virus (RAS), nuclear factor kappa B (NFkB), transforming growth factor beta (TGF-β), and losing BReast CAncer gene 1 (BRCA1) expression [11]. The transformed fibroblasts (allies CAF) behave similarly to myofibroblasts in that they can initiate the cell divisions themselves (scaring process like in myofibroblast). However, it differs in activating carcinogenesis rather than apoptosis [12]. Tumor-signaled myofibroblasts secrete various cytokines that activate the cancer cells to attract other cells and reduce the immune surveillance or to form a mechanical barrier for reducing drug concentrations within tumors [13].

Earlier studies showed that the extracellular matrix (ECM) also plays a vital role in alleviating the stromal transforming efficiency to the maximum and reverting the chromosomal alteration meant for regular cell physiology [14]. However, the fibroblasts in benign environments inhibit tumor development. The graded cancer cell environment during the process of carcinogenesis converts those fibroblasts into CAF [15]. The nature and the structure of stromal cells in benign microenvironments significantly differ from normal stromal cells. These modified cells are associated with the cancer microenvironment. Hence, stroma has a unique driving mechanism that usually alters the CAF [16]. The previous studies showed that the conversion of fibroblast in favoring cancer growth is one directional. Once converted, CAF at the TME could not reverse into the benign types of stromal fibroblast. The cancer epithelia play a prominent role in cancer pathogenesis.

Recently, a study showed a coevolution of the epithelia and fibroblast to favor tumor development [17]. The epigenetic variations contribute sustainably to tumor growth by stimulating severity at several surrounding cells from simple tumor to metastasis [18]. Several clinical trials are used to identify and assess the position and stages of the CAFs for diagnosing the tumor stages in patients (Table 1). Thus, TME and CAF provide excellent openings and novel chances for identifying valuable targets for cancer drug discovery. The TME consists of several elements, including blood vessels, immune cells, extracellular matrix, and CAFs. These elements interact with cancer cells and are essential for the growth and development of tumors. TME affects immunological response, therapeutic response, and behavior of cancer cells. Through the secretion of growth factors, ECM remodeling, and inflammatory promotion, CAFs enable tumor growth, invasion, and metastasis.

**Table 1:** CAFs serve as diagnosing tools for ensuring cancer stages. CAF: Cancer-associated fibroblasts; TME: Tumor microenvironment; NA: Not applicable; FAP: Fibroblast activation protein; EUS: Endoscopic ultrasound; FNB: Fine needle biopsy; PDAC: Pancreatic ductal adenocarcinoma; FAPI: Fibroblast activation protein inhibitors; PET: Positron emission tomography; CT: Computed tomography; CRC1: Colorectal cancer; OSCC: Oral squamous cell carcinoma; MMT: Mesothelial-mesenchymal transition; LCM: Laser capture microdissection

Nature of the Trial	Method	Phase	Identification Number of Clinical Trials
Assessing the impact of cross-talking between the lung CAF co-cultured with fibroblast isolated from normal volunteers in TME	This study would reveal the scientific intervention behind the mechanism of cross-talking	NA	NCT02161523
Histological findings from patients with epithelial ovarian cancer who had radioactive 68Ga-FAPI-04 and 18F-FDG PET/CT in comparison	Diagnosis intervention for comparison with the histology	II	NCT04504110
Analysis of the characteristic feature of 68Ga-FAP-2286 tracer binds to FAP, a transmembrane protein in CAFs.	Diagnosis intervention	I	NCT04621435
EUS-guided FNB results from patients with PDAC were analyzed for pharmacotyping.	Diagnosis intervention	NA	NCT05196334
Analyzing the diagnostic value of 68Ga-FAPI PET/CT in tumor	68Ga-FAPI PET/CT is an inhibitor and binds to CAFs. Analyzing its role would reveal essential points for effective diagnosis.	NA	NCT05034146
Employing the imaging agent (F-18) fluorodeoxyglucose to apply radionuclide-labeled FAPI, specifically to FAP proteins in CAFs (18F-FDG)	Diagnosis intervention	NA	NCT04554719
Comparative analysis of 68Ga-FAPi-46 PET/CT bound to CAFs in various cancer types, including bladder carcinoma, cervical carcinoma, cholangiocarcinoma, hematopoietic and lymphoid cell neoplasm, hepatocellular neoplasm, malignant adrenal gland neoplasm, malignant brain neoplasm, malignant pleural neoplasm, malignant skin neoplasm, malignant solid neoplasm	Prospective Exploratory PET/CT	I	NCT04459273
FAPI PET/CT image analysis on CRC1 (FAPI-CRC1)	FAPI PET or CT	NA	NCT05209750
Imaging of CAFs in OSCC (FAPI-OSCC) using 68Ga-FAPI	FAPI PET or CT	NA	NCT05030597
Imaging of CAFs in PDAC (FAPI-PDAC) using 68Ga-FAPI	[68Ga]FAPI-46	II	NCT05262855
To analyze the stages of colorectal carcinomatosis (MMT) using MMT cells and CAFs	Sampling peritoneal tissue using LCM and gene expression analysis	NA	NCT03777943
Analyzing the safety and tolerability of 177Lu-FAP-2286 in solid tumors	68Ga-FAP-2286 and 177Lu-FAP-2286	I and II	NCT04939610
To categorize the Hepatic Tumor based on the expression of CAFs	Prospective study	NA	NCT02587793

## Review

CAFs: heterogeneity in population and their origin

The CAFs seemed to have a different population of their subsets that rose from the heterogenic signaling from the TME based on the local sources [19]. The activation and the differentiation strongly depend on chemical regulators such as platelet-derived growth factor (PDGF), TGF-β, epidermal growth factor (EGF), and fibroblast growth factor 2 (FGF-2). The interleukin-1 beta (IL-1β) plays a significant role in CAF activation and leads to respective secretomes that favor tumor progression [20]. The transdifferentiation of blood cells, epithelial cells, pericytes, and adipocytes into CAF is also possible at TME. Similar activation was recorded in breast cancer (BC) [21]. In addition, the bone marrow-derived mesenchymal cells and tumor-associated mesenchymal stem cells (TA-MSC) also differentiate into CAFs at TME. Similar to the normal ones, the CAFs also require their counterparts but differ in their activating ability [22]. Their efficiency is mainly determined by the stimuli from the TME, such as hypoxia, oxidative stress, and other growth factors. Besides, tumor cells and immune cells also contribute to their unregulated activation [23]. The CAF subsets are predominately varied and classified based on their alpha-smooth muscle actin (α-SMA) expression pattern [24].

The cancer cells acquire drug resistance and unregulated proliferation ability very rapidly and remain a major challenge for chemotherapies. Several strategies are used in modifying the tumor-associated functions of the CAFs, a few of them reducing the immune suppressive activity for enhancing anti-carcinogenic activity [25]. Fibroblast activation protein (FAP) depletion, increasing the vitamin A concentration, activation of vitamin D receptor, primary sclerosing cholangitis (PSC) deactivation, C-X-C motif chemokine ligand 12 (CXCL12) blocking, TGF-β inhibition, and inhibiting the Janus kinase/signal transducers and activators of transcription 3 (JAK/STAT3) pathway are a few of the strategies used in CAF's targeted therapies. In all instances, these drug-developing programs aimed at rewiring the chemical signal between the CAFs, surrounding tissues, and the cancer cells. Thus, CAFs offer a successful opportunity for drug discovery programs. Several studies have shown that CAFs are very stable and have fewer tendencies to develop resistance than cancer cells [12-20]. Moreover, they occupy spatially defined surroundings in TME and have higher proximity to the blood vessels. These instances make them a prominent candidate for drug discovery. The current study focuses on the recent advances in CAF signaling and their application in cancer drug discovery.

Tumor-promoting properties of CAFs and drug discovering strategies

The chemical conversation between fibroblast and epithelial cells in a tumor environment is an efficient strategy for inhibiting cancer growth. Several studies showed that CAF positively and negatively regulates the oncogenes and anti-apoptotic factors in cancer sites [26]. Neri et al. (2017) showed that CAFs increased the expression of PDGF-B in cancers and contributed to the aggressive behavior of tumors [27]. Their study showed that the CAFs during the epithelial-mesenchymal transition (EMT) increased the ECM remodeling through PDGF-B expression. Further, they analyzed its role in patients with lung adenocarcinomas (N=442). They found that PDGF-B accelerated the disease recurrence and poor prognosis in the presence of higher CAF expression. Kim et al. (2022) showed that GLIS1 increased the aggressive property of osteosarcoma (OSC) in patients, particularly over-expressed in high-grade OSCs. GLI-similar protein (GLIS1) is a type of Kruppel-like protein, usually found at higher levels in embryos and unfertilized eggs. These factors can induce pluripotent cells (iPS) from the somatic cells. They clearly showed that CAFs contained elevated levels of mGLIS1 (mRNA) and increased levels of cytokines such as TGF-β, fibroblast growth factor-1 (FGF-1), VEGFA, interleukin 6 (IL-6), CXCL12, and TGF-α than normal cells. FGFβ could either increase the CAF density or vice versa. Thus, FGFβ along with CAFs modify the tumor phenotype and aggressiveness [28-30]. In ovarian TFI SKOV3 cells, Sun et al. (2017) showed that CAFs (isolated from the patients) increased the phosphorylation of the FGF-1 and induced the mitogen-activated protein kinase (MAPK)/extracellular signal-regulated protein kinase pathway [28]. Eventually, its increase also induced the Snail1 (EMT pathway) and MMP3 expression [28]. CAF was shown to modulate the HIF1α/VEGFA axis through the elevated level of miR-320a in endometrial cancers [31].

As discussed earlier, the CAFs greatly vary with α-SMA expression and showed higher expression (up to 98%) at invasive front and intratumoral stroma regions [32]. CAF predominately enhances cancerous growth using regulating different growth factors and TME and offers a great opportunity for cancer drug discovery. The enhanced aggressiveness of tumors is partly attributed to elevated expression of α-SMA [32]. The function of CAFs is contingent upon the expression of S100A4, podoplanin, and α-smooth muscle actin. Their expression directly affects patient survival and has a significant impact on it [33]. One important factor that significantly affects a patient's likelihood of survival is FAP [34]. Tumor growth is aided by the activation of PDGF alpha/beta (PDGFRα/β) [35]. In the TME, Tenascin C contributes to the enhancement of the EMT [35,36]. NG2 neuron glial antigen has a particular kind of surface marker for CAF in assessing the aggressiveness of the tumor [36]. Compared to CXCL12 generated by normal fibroblast cells, CAF isolated from the esophagogastric junction (AEG) cancer showed enhanced invasive and metastatic capabilities. The potential significance of CXCL12 in inducing malignancy is shown by its higher level in these CAFs [37,38]. The regulation of CAFs in TME is highly complex. Several growth factors/signaling proteins enhance the CAFs at the initial stages of carcinogenesis. In later stages, CAFs were shown to regulate them according to the cancer signals [38].

Osteopontin (OPN) is a type of chemokine-like protein that has an essential role in regulating the cell proliferation and invasion of many cancer types. A recent study showed that CAF controls the OPN through the IL-6 in head and neck cancers [39]. They also showed that the cancer growth progression was based on the OPN-NFkB signaling pathway. Minnelide (a prodrug currently in clinical phases II and III) showed increased apoptotic protein expression in gemcitabine-resistant pancreatic cancer [40]. Dauer et al. (2018) showed that inactivating the CAF to prevent cancer stromal cross-talking resulted in the inhibition of oncogenic proteins. They used minnelide for inhibiting the TGF-β pathway in CAFs and showed that the fibroblast had undergone into the quiescent, nonproliferative state. This inhibition showed higher inhibition of stromal CAF functions [41].

MicroRNAs (miRNAs) are a kind of shortened noncoding RNAs that regulate a variety of gene expressions, especially oncogenes and tumor suppressors. miR-106 directly regulates cell proliferation, invasion, migration, and metastasis of several cancer types [42]. Fang et al. (2019) reported that CAF plays a role in chemoresistance in pancreatic cells against gemcitabine by enhancing the miR-106 [43]. The protein Tenascin C is a well-known ECM modulator that is strongly associated with CAFs in several studies. 131I-m81C6, a Tenascin C inhibitor, has been shown to ameliorate ECM stiffness and lesser CAF differentiation [43]. Using transmission electron microscopy (TEM) and western blot, they showed that CAFs directly transferred the miR-106 into cancer cells using exosomes and elevating the expression of anti-proliferative and apoptotic proteins, tumor protein 53-induced nuclear protein 1 (TP53INP1). WNT2 acts as a pro-angiogenic factor, and its expression increases the angiogenesis in a variety of endothelial cells (ECs). Several cancers were shown to have elevated expression of WNT2 about metastasis. WNT2 was also shown to selectively enhance the CAF in TME for facilitating colon cancer progression [44]. Daniela et al. (2020) showed that the knockdown of WNT2 inactivated the CAFs and reduced the angiogenesis and metastasis behavior [45]. They showed that the secretome of the CAF contained higher levels of pro-angiogenic proteins such as granulocyte colony-stimulating factor (G-CSF), Angiopoietin 2 (ANG-2), placental growth factor (PGF), and IL-6 using mass spectrometry and cytokine arrays. Several studies have focused tumorigenic properties of CAFs, either directly (through CAF reduction or reprogramming to a normal fibroblast phenotype) or indirectly over the last decade. Clinical trials have been developed from some of these investigations (Table 2).

**Table 2:** List of clinical trials focusing on the therapeutic values of CAFs Am80: A retinoic acid receptor agonist; pCAF: Cancer promoting cancer-associated fibroblasts; rCAF: Cancer restraining cancer-associated fibroblasts; MCT4: Monocarboxylate transporter 4; NAC: N-acetyl cysteine; BC: Breast cancer; PVHA: Pegvorhyaluronidase alfa; HA: Hyaluronan; ECM: Extracellular matrix; PEGPH20: PEGylated hyaluronidase

Nature of the Trial	Intervention	Phase	Identification Number of Clinical Trials
The type converting agent, Am80, that converts pCAFs into rCAFs (i.e., pCAf promotes cancer and rCAF restrains the cancer progression)	Am80 (Tamibarotene, MIKE-1)	I and II	NCT05064618
Analyzing the effect of NAC in BC patients (stage 0/1)	The change in expression of caveolin-1 and MCT4 of the CAFs in pre- and post-therapy patients treated with NAC	I	NCT01878695
Applications of PVHA (PEGPH20) bind and eliminate the HA from ECM and increase the vasculature. In addition, avelumab application inhibits the checkpoint inhibitors further.	PEGPH20, recombinant avelumab	I	NCT03481920

Pure CAF-targeting medications rarely succeed due to the dual role of CAFs in TME, given cancer-promoting activities. In the next section, we concisely reviewed the role of CAFs in modulating the components of extracellular matrix (CAM). In the TME, overexpression of tenascin causes fibroblasts to become activated and differentiated [45].

Role of CAFs in modulating ECM

The ECM, which is a non-cellular stromal component, has an impact on many cellular activities. It is made up of glycosaminoglycans, structural proteins, and matricellular proteins that help cells communicate, adhere, and move [46]. Tumors change the density and content of the ECM. Tumor growth and progression are aided by the changes toward rigidity and deterioration. The greatest contribution to ECM stiffness and degradation is CAF [47]. CAFs regulate multiple microRNAs (miR-21-5p, miR-133a-3p, and miR-1-3p) to increase the production of laminins, which contributes to the destruction of the ECM. In comparison to other locations, the expression of laminins was significantly higher at deeper tumor sites, suggesting a localized effect on the TME [47]. Thus, CAFs could produce a variety of ECM-modulating factors that change the nature of cancer stroma. Laminin is one among them, and several studies showed a direct correlation between CAF differentiation and increased laminin expression.

Cavaco et al. (2018) showed that CAFs achieved ectopic deposition in the TME by producing the principal laminin receptor, α3β1 integrin, using the spheroid culture system of pancreatic duct adenocarcinoma AsPC-1 cells [48]. They also found lesser invasion with integrin α3-deficient CAFs. Among other multicellular proteins [49], periostin (POSTN) is a key factor for regulating cellular proliferation, and its expression is strongly correlated with tumor progression. Yamauchi et al. (2021) showed the correlation between CAFs and periostin in patients with esophageal squamous cell carcinoma (ESCC) [50]. Yamauchi et al. (2021) showed the correlation between CAFs and periostin in patients with ESCC [50]. They also showed that the elevated expression of POSTN was associated with a shorter survival time and disease relapse.

Yu et al. (2018) showed that the CAFs with protein tyrosine kinase 7 (PTK7) increased the cancer aggressiveness in head and neck cancer [51]. Their study showed that the PTK7 enhanced tumor growth through the Wnt/β-catenin pathway. The co-immunoprecipitation assay showed that POSTN secreted by CAFs functioned as an upstream ligand for elevated PTK7. Further, they also showed that PTK7 increased the stiffness of the cancer stroma. Matrikines, peptides derived from extracellular matrix protein fragmentation, have different biological activities compared to the parent protein [52]. Several studies showed that the CAFs influenced the expression of those bioactive proteins [53]. Agrin is a basement membrane (BM)-associated proteoglycan and has an essential role in ECM modulation. It enhanced the EMT and its loss led to the phosphorylated focal adhesion kinase (pFAK) localization during the cell division [54]. Samain et al. (2021) showed that the CAFs downregulated the agrin using their secretome analysis [55]. Hyaluronan (HA) is a kind of extracellular polysaccharide that helps in cell motility and plays a prominent role in initiating carcinogenesis [56]. By performing various tasks, HA in the TME induces cancer and its progression [57]. The involvement of HA in activating CAFs in pre-malignant and malignant stroma, as well as enhancing invasion by increasing the mobility of both CAFs and tumor cells, makes it easier for them to invade further [58]. Thus, HA increases the chances of circulating CAFs (cCAFs), and these cCAFs serve as a precursor for metastasis initiation [59].

Fibronectin, a glycoprotein, helps in cell adhesion, mobility, development, and tissue repair. Different factors involved in modulating their role in pathogenesis and CAFs have been shown to increase the anisotropic orientation by increasing the nonmuscle myosin II, PDGF-α, and α5β1 integrin [60]. Huang et al. (2021) showed that regulation of TGF-β1 and fibronectin could be a promising strategy for inhibiting CAF activation and oral cancer types [61]. Laminin 322, an ECM protein, is important in maintaining cell adhesion. So, if it is not functional, extracellular proteolysis will be affected. This matrix protein is related to CAFs [62]. MicroRNAs (miR-21-5p, miR-133a-3p, and miR-1-3p) are regulated by CAFs, which cause an increase in laminin synthesis. Consequently, this contributes to the breakdown of the ECM. Notably, compared to other locations, the expression of laminins demonstrated a much higher level at deeper tumor sites, suggesting a localized influence inside the tumor microenvironment [62]. CAFs induce the laminins for degrading ECM by regulating several microRNAs (miR-21-5p, miR-133a-3p, and miR-1-3p). [47,62,63]. Tenascin over-expression can lead to activation and differentiation of fibroblast [64,65]. CAFs exert strong regulation over periostin, and higher expression levels are linked to more aggressive tumors [66,67]. Matrikines are impacted by CAFs either directly or indirectly [68]. HA improves motility and aids in the creation of a niche conducive to metastasis by mediating contact between CAFs and tumor cells. In the TME, HA plays a key role in the CAF-driven EMT and the subsequent development of tumor clusters [69-71]. Fibronectin significantly affects cellular migration patterns in the tumor microenvironment by actively changing the migratory behavior toward cancer cells. This change in migration emphasizes how fibronectin dynamically shapes cell-to-cell contacts in the setting of cancer progression [71,72].

The role of fibronectin in the modification of the migration towards the cancer cells is well documented [73]. Desmoplasia, a stiffening stroma that encourages cancer cells to behave aggressively, is linked to solid tumors, particularly those arising from glandular epithelium. CAFs are renowned for constantly remodeling the ECM, which usually results in increased stiffness and desmoplasia [73]. CAFs are characterized by ongoing ECM remodeling, which often results in the ECM becoming stiffer due to the excessive deposition of type I and type III collagens (Col-I, III) and the breakdown of type IV collagen [74,75].

The most significant components of the ECM are collagens. While the BM breach and type IV collagen turnover are well-known components of carcinogenesis, little is known about the effect of stromal collagens on tumorigenesis [76]. Collagens are the most abundant proteins in the ECM, accounting for up to 30% of the human body's total protein mass [77]. Tensile strength is achieved by arranging collagen fibers in a relaxed meshwork surrounded by proteins such as elastin and glycoproteins [78]. Thus, modulating the expression of collagen-related proteins is a promising strategy for inactivating the CAFs and achieving tumor inhibition.

Today, there are 28 different types of collagen, each of which contributes to the unique ECM composition seen in different tissues. The 28 collagens have been divided into many subgroups, the most well-studied of which are the fibrillar-forming and network-forming collagens [79]. This extra deposition ensures the self-sustainability of the CAFs [80]. TGF-β, PDGF, and FGF-2, among others, released by malignant cells attract the nearby fibroblast and turn them into CAF phenotypes. This method converts up to 80% of normal fibroblasts into CAFs [81]. Desmoplasia, a stiffening stroma that encourages cancer cells to behave aggressively, is linked to solid tumors, particularly those arising from glandular epithelium. CAFs are renowned for constantly remodeling the ECM, which usually results in increased stiffness and desmoplasia [73]. Integrin alpha-11 (ITGA11) is a protein found in mussels and dimerized with β1 integrin for activating collagen fibers. ITGA11 knockdown attenuated the myofibrillar differentiation and hedgehog pathway [82]. Iwai et al. (2021) showed that ITGA11, collagen type XI alpha 1 (COL11A1), and ITGA11 were the most expressive proteins in CAFs isolated from patients with non‐small cell lung cancer (NSCLC) [83]. They also found that the elevated level of ITGA11 was correlated with cancer stroma and higher CAFs through the extracellular signal-regulated kinase 1/2 (ERK1/2) pathways. Yuan et al. (2015) showed that the knockdown of β1-integrin in Michigan Cancer Foundation-7 (MCF-7R) cells brought the chemosensitivity towards tamoxifen and inhibition of CFA activation [84]. Thus, the inactivation of CAFs also brings chemosensitivity in cancer cells.

Role of CAFs in regulating chemoresistance

Chemoresistance prediction, which can aid in clinical decision-making about proposed treatment protocols, is one of the prognostic implications of CAFs. On the one hand, this is because CAFs have a positive connection with desmoplasia [85]. Gemcitabine, the chemotherapeutic mainstay of therapy for pancreatic ductal adenocarcinoma (PDAC), is innately resistant to CAFs. Furthermore, CAFs treated with gemcitabine produce more exosomes, which are extracellular vesicles. In recipient epithelial cells, these exosomes enhanced the chemoresistance-inducing factor Snail, which promotes proliferation and drug resistance. Treating gemcitabine-exposed CAFs with GW4869, an inhibitor of exosome release, lowers survival in co-cultured epithelial cells, indicating that CAF exosomes play a key role in chemotherapeutic drug resistance [86]. Microfibril-associated protein 5 (MFAP5) is used in cell adherence and development. The elevated secretion of MFAP5 from CAFs led to drug sensitivity and cell migration in ovarian and pancreatic cancer. Monoclonal antibody (Mb) of MFAP5 inhibited collagen production and suppressed the intratumoral microvessels [87]. They also showed that the cells exhibited higher sensitivity towards paclitaxel.

As discussed previously, the microRNA inhibition on the CAF proteome brought down chemoresistance in many cancer types. Tanaka et al. (2015) showed that the normal fibroblast varying with CAFs and the cancer-favoring miRNAs were highly expressed in CAFs [88,89]. They showed that, among highly expressed 18 miRNAs, normal fibroblasts are transformed into CAFs by miR-27a/b, and it also increased drug sensitivity in esophageal cancer. Shan et al. (2021) showed that the inhibition of miR-148b-3p inhibited the EMT activation, metastasis, and chemoresistance in xenograft bladder cancer mouse model through the Wnt/β-catenin pathway [90]. Cytokines produced from CAFs strongly maintain the TME in favor of tumor growth [90]. The activity of bone morphogenetic proteins (BMPs), BMP2 and BMP4, is inhibited in a dose-dependent manner by cytokine, gremlin 2 (GREM2), which affects BMP family signaling. BMP family members play a role in the regulation of embryonic morphogenesis. GREM2 also inhibits the reduction of progesterone synthesis in granulosa cells caused by BMP4 [91]. CAFs decreased the sensitivity towards the taxane in prostate cancer. Shan et al. (2020) showed that inhibiting the miR-423-5p from the CAF exosome decreased the GREM2 and drug resistance [92].

Role of TQ on TME

For centuries, the black seed,* Nigella sativa* has been used in herbal medicine because it is the source of many herbal medicines. It has medicinal properties such as anti-neoplastic, anti-inflammatory, anti-oxidant, anti-asthmatic, analgesic, anti-pyretic, anti-hypertensive, and anti-microbial [93]. In *Nigella sativa*, there are different types of bioactive compounds. Recently, TQ has attracted the attention of researchers, which is one of the biochemicals present in *Nigella sativa*. It is an herbal drug for the treatment of different diseases and can prevent particular diseases. So, it is used in herbal medicine in many nations of the world [94]. Specifically, TQ has shown anticancer properties in BC, prostate cancer, bone cancer, gastric cancer, bladder cancer, colon cancer, and lung cancer [95].

TQ has antiproliferative effects, which have been demonstrated on different cancer cell lines [96]. Different roles of TQ include lowering VEGF, reducing the activity of angiogenesis, increasing serum interferon‐gamma (INF-γ) levels, and evidently, the immunity responded to T helper1 in BC models [97]. It has also been reported that by targeting cyclin E, cyclin D1, and p27 proteins, TQ caused cell cycle arrest, suppressing progression from G1 to S phase [98]. Studies show that TQ can induce apoptosis [99].

CAFs in tumor angiogenesis and cancer progression

In determining cell function and its phenotype, fibroblasts are needed as they set scaffold for the cells [100]. CAFs increase angiogenesis and cancer progression [101]. They are a significant source of tumor VEGFA and support tumor angiogenesis in a VEGFA-independent manner [102,103]. CAF-derived PDGFC maintains angiogenesis by further CAFs to get stimulated and release pro-angiogenic growth factors, such as FGF-2 and OPN [104].

CAFs enhance certain cancer cell migration, invasion, and proliferation and participate in the EMT process via the Wnt/β-catenin pathway [105]. Other studies found that CAFs with high expression of leucine-rich repeat-containing protein 15 (LRRC15) increase cell migration and invasion by influencing the Wnt/β-catenin signaling pathway. These studies were conducted in MDA-MB-231 and MDA-MB-468 triple-negative BC (TNBC) cell lines [103-105].

TQ as a bioactive molecule interfering with oncogenic factors in TME

A study showed that TQ inhibited PDGF-BB, which resulted in lowering the proliferation and migration of vascular smooth muscle cells.

**Figure 2:**
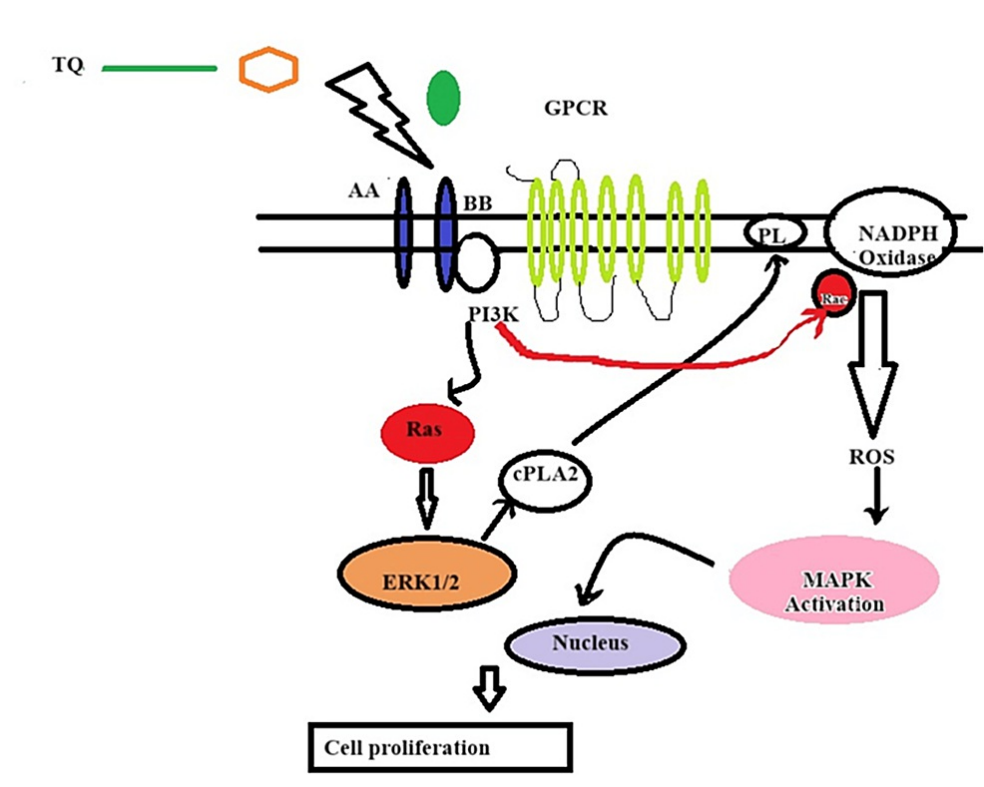
Simplified mechanism of TQ TQ, a bioactive compound found in black seed oil, demonstrates inhibitory effects on PDGF-BB. PDGF-BB is a key signaling molecule implicated in various cellular processes, including cell proliferation and differentiation. TQ's capacity to inhibit PDGF-BB suggests its potential to disrupt pathways associated with abnormal cell growth and tissue remodeling. By impeding the actions of PDGF-BB, TQ may exert anti-proliferative and anti-fibrotic effects, highlighting its therapeutic potential in conditions in which dysregulated PDGF signaling contributes to pathogenic processes, such as in certain cancers and fibrotic disorders. TQ: Thymoquinone; PDGF-BB: Platelet-derived growth factor-BB Image credit: Meenakshi Sundaram

The study was conducted by authors both in vitro and in vivo [106]. TQ concentration-dependently inhibited several growth factors, such as EGF and VEGF, the main CAF sources, in BC cell lines in Bagg Albino (Balb/C) mice [107]. In in vitro studies on MDA-MB-231 cancer stem cells, TQ inhibited Wnt3a and phosphatidylinositol-3 kinase (PI3K) and blunted the stimulatory effects of VEGF, EGF, and FGF. The study was further confirmed by demonstrating a lack of cellular response to pro-angiogenesis factors. These data suggest that targeting CAFs is being considered to control TNBC using TQ [108]. A study conducted on the effect of TQ on human astrocytoma cells and in Jurkat cells (T lymphoblastic leukemia cells) shows that there is degradation of α/β tubulin and was associated with upregulation of tumor suppressor p73 with apoptosis [109]. The simplified mechanism of TQ is given in Figure 2. TQs are flavonoids; they are polyphenolic compounds further subdivided into six groups [110]. These include isoflavonoids, flavanones, flavanols, flavonols, flavones, and anthocyanidins [111-113].

TQ concentration-dependently inhibited several growth factors, such as EGF and VEGF, the main CAF sources, in BC cell lines in Balb/C mice [108]. In in vitro studies on MDA-MB-231 cancer stem cells, TQ inhibited Wnt3a and PI3K and blunted the stimulatory effects of VEGF, EGF, and FGF. The study was further confirmed by demonstrating a lack of cellular response to pro-angiogenesis factors. These data suggest that targeting CAFs is being considered to control TNBC using TQ. A study conducted on the effect of TQ on human astrocytoma cells and Jurkat cells (T lymphoblastic leukemia cells) shows that there is degradation of α/β tubulin and was associated with upregulation of tumor suppressor p73 and apoptosis.

Mechanism of action of flavonoids

Flavonoids have anti-inflammatory and anticancer activities and they enhance the immune system [114]. Flavonoids have been shown to possess a wide variety of anticancer effects. They modulate reactive oxygen species (ROS) scavenging enzyme activities, participate in arresting the cell cycle, induce apoptosis and autophagy, and suppress cancer cell proliferation and invasiveness [115,116]. Flavonoids have dual action regarding ROS homeostasis. They act as antioxidants under normal conditions and are potent pro-oxidants in cancer cells, triggering the apoptotic pathways and downregulating pro-inflammatory signaling pathways. Anticancer effects include nuclear receptors, the aryl hydrocarbon receptor (AhR), kinases, receptor tyrosine kinases (RTKs), and G protein-coupled receptors [117]. However, flavonoids have poor drug delivery and poor bioavailability. Flavonoid bioavailability is low due to restrictions in absorption, modifications throughout the gastrointestinal tract caused by microorganisms, chemical and mechanical effects, and rapid excretion. Flavonoids possess a basic 15-carbon flavone skeleton, C6-C3-C6, with two benzene rings (A and B) linked by a three-carbon pyran ring (C). The position of the catechol B-ring on the pyran C-ring and the number and position of hydroxy groups on the catechol group of the B-ring influence the flavonoids’ antioxidant capacity. Glycosylation enhances solubility, distribution, and metabolism by facilitating transport through the membrane, and methylation increases the entry of flavonoids into the cells and protects them [118].

Flavonoids contain a common phenylchromen-4-one scaffold, which can be substituted with a phenyl ring at C2 or C3 to give the flavone and isoflavone backbone structure. Further modifications occur at C4 (a ketone group), C2-C3 (saturated or olefinic), plus hydroxy or methoxy substituents on the phenylchromen-4-one and phenyl rings, resulting in the formation of flavanones, flavanols, flavonols, flavones, anthocyanidins, and isoflavones. The effects of these compounds have been attributed, in part, to their activities as antioxidants, antimicrobial and antiviral activities, radical trapping agents, and inhibitors of key enzymes/factors such as cyclooxygenases and acetylcholinesterase. Flavonoids act as antioxidants by donating hydrogen ions [119].

Flavonoids as kinase inhibitors

Many studies have reported that flavonoids bind with many proteins and modify their activities. Studies also show the interactions of flavonoids with different kinases and kinase-dependent signaling [120].

Among the flavonoids, genistein was the first flavonoid to be recognized, and it is an RTK inhibitor [121]. Moreover, it inhibits autophosphorylation of the epidermal growth factor receptor (EGFR) and acts as a non-competitive inhibitor of histone H2B [122]. Further studies indicated that genistein and other different flavonoids inhibit many kinases. They have direct action against many proteins. This was confirmed by studies of flavonoids and their structural importance. Hou and Kumamoto concluded that flavonoids bind with many kinases, showing both similarities and differences concerning their interactions with one or more sites in multiple kinases [123]. For example, myricetin binds the ATP pocket of Akt1, MAPK kinase 4 (MKK4), Fyn, P13Kγ, p38MAPK, and JNK3. Myricetin also binds MAPK kinase 1 (MEK1) and Janus kinase 1 (JAK1). Myricetin is also found to be binding with sites of both p38MAPK and JNK3 in modeling studies. These results are in line with the docking studies where the authors found that flavonoids bind the ATP sites of JNK3 (acacetin, velutin, chrysoeriol, luteolin, and myricetin), and the β ring of myricetin is oriented in the binding site in the opposite direction compared to the other flavonoids [124]. It was also found that structure-dependent binding of 16 flavonoids to three acidophilic Ser/Thr protein kinases. These kinases are Golgi apparatus casein kinase (G-CK), CK1, and CK2 [125]. It was observed that flavonoids (≤ 40 µM) inhibit G-CK in a minimal range. More structure-dependent inhibitory effects of flavonoids on CK1 activity were also noted. In contrast, at least six flavonoids inhibited CK2 with IC50 values ≤ 1 µM and the presence of both 7- and 4΄-hydroxyl groups was a common structural feature of the active flavonoids, which appear to occupy the ATP binding pocket. This is observed as an underlying mechanism of flavonoids for inhibiting multiple tyrosine kinases. However, despite the extensive evidence showing that flavonoids inhibit multiple tyrosine kinases, the clinical applications of these compounds as targeted kinase inhibitors are minimal.

Flavonoids can bind directly to some protein kinases, including Akt/protein kinase B (Akt/PKB), Fyn, JAK1, MEK1, phosphoinositide 3-kinase (PI3K), an MKK4, Raf1, and zeta chain-associated 70-kDa protein (ZAP-70) kinase, and then alter their phosphorylation state to regulate multiple cell signaling pathways in carcinogenesis processes. The MAPK signaling pathway plays an important role in cancer cell proliferation and survival. MAPKs’ protein kinases, MEK1/2, serve as important targets in drug designing against cancer. The natural compounds’ flavonoids are known for their anticancer activity. Flavonoids can act as protein kinase inhibitors, which can be considered for cancer chemoprevention. These observations are pointed out by authors who did their studies using direct binding and molecular modeling [126].

Interaction of flavonoids within neuronal signaling pathways

In neurons, flavonoids act on different protein kinase pathways and lipid kinase signaling cascades, such as PI3K/Akt, protein kinase C, and MAPK. In a nutshell, their activity can be summarized as their ability to modify the action of kinases and phosphatases. They also have the binding capacity on ATP binding sites of different enzymes and receptors involved in kinase pathways so that they activate the state of target molecules and/or modulate gene expression [127].

2'-hydroxyflavanone (2'-HFa) inhibits tumor cell proliferation and restricts tumor growth. This mechanism binds to the Ral-interacting protein RLIP, which is implicated in the transport of glutathione conjugates [127].

Interaction of flavonoids with G-protein-coupled receptors

**Figure 3:**
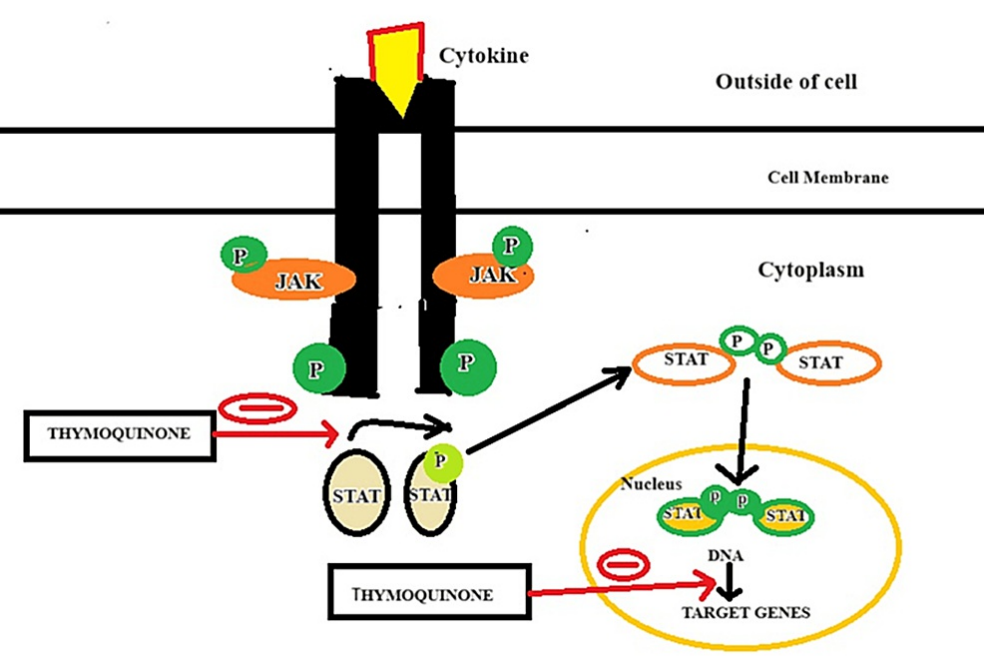
TQ by downregulating JAK/STAT pathway TQ exhibits anticancer potential by downregulating the JAK/STAT pathway and suppressing CAFs. By inhibiting the JAK/STAT signaling cascade, TQ disrupts aberrant cellular proliferation and survival mechanisms associated with cancer. Moreover, its ability to target CAFs, which play a crucial role in tumor growth and progression, contributes to an unfavorable microenvironment for cancer cells. This dual action underscores TQ's promising therapeutic role in impeding cancer development by disrupting key signaling pathways and modulating the tumor stroma by inhibiting cancer-promoting fibroblasts. TQ: Thymoquinone; JAK/STAT: Janus kinase/signal transducers and activators of transcription; CAFs: Cancer-associated fibroblasts Image credit: Meenakshi Sundaram

Flavonoid interacts with G-protein-coupled receptors. Some of the examples are given in Table 3 and Figure 3.

**Table 3:** Flavonoid interactions/modulation of G-protein-coupled receptors EGCG: Epigallocatechin gallate; PTHR1: Parathyroid hormone receptor 1; JAK/STAT: Janus kinase/signal transducers and activators of transcription

Flavonoid Type	Receptor	Mechanism	References
EGCG (antagonist)	EP1 (prostaglandin receptor)	Increased the ratio of Bax/Bcl-2	[128]
Acacetin	5-HT_1A_	Depresses monoamine oxidase	[129]
Quercetin (ant)	PTHR1	Reduction of PTHR1 protein expression	[130]
Multiple	Thromboxane receptors	Contribution of C7 and C8 carbons in the A ring, γ pyrone structure conjugated with a double bond between C2 and C3 carbons in the ring, and C2`, C3`, and C4` carbons in the B ring as the structural determinants that create the active flavonoid skeleton in thromboxane A2 specific membrane receptor blockade.	[131]
Cytokine receptors/growth factor receptors	Quercetin	Induces p53 activation resulting in upregulation of Bax and downregulation of Bcl-2 in tumor cells. JAK/STAT pathways.	[132]

Whereas, TQ acts via cytokine/growth factor receptors, downregulating JAK/STAT pathway [133].

Flavonoids and AhR

**Figure 4:**
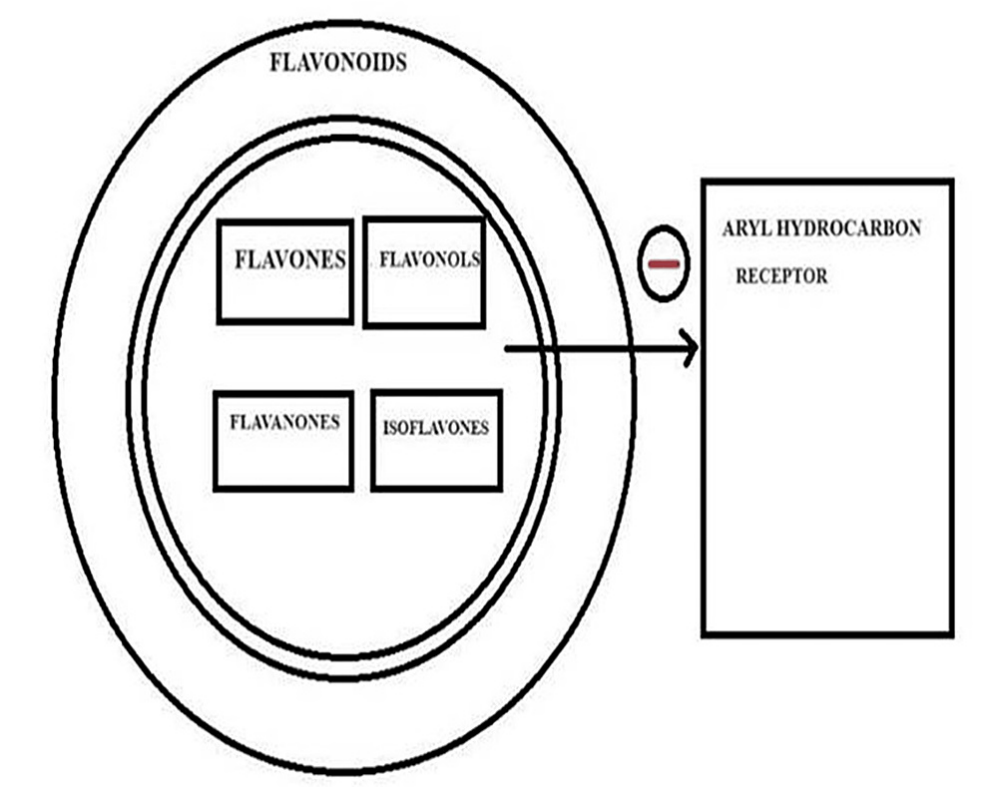
Action of flavonoids on AhR Some flavonoids function as AhR ligands, activating their transcriptional activity and influencing gene expression. Others act as antagonists, impeding AhR activation. This interaction modulates cellular processes, impacting xenobiotic metabolism, immune regulation, and inflammation. The complex relationship between flavonoids and AhR suggests potential health benefits, such as anti-cancer effects. However, outcomes are context-dependent, varying with flavonoid type and cellular conditions. Understanding these interactions enhances insight into the role of flavonoids in health and their potential applications in disease prevention and management. AhR: Aryl hydrocarbon receptor Image credit: Meenakshi Sundaram

The AhR is a basic-helix-loop-helix transcription factor that forms an active nuclear heterodimer with the AhR nuclear translocator (ARNT) protein to activate gene expression (Figure 4).

Extensive structure-activity studies demonstrate that different classes of flavonoids exhibit AhR activity, as evidenced by their induction of AhR-responsive CYP1A1 gene expression in cell lines and animal models. The 4΄,5,7-trimethoxyflavone was inactive as an inducer whereas the magnitude of 4΄,5,7-trimethoxy-isoflavone was similar to that observed for 2,3,7,8-tetrachlorodinezo-p-dioxin (TCDD) in Caco2 cells.

There can be AhR-responsive CYP1A1, CYP1B1, and UGT1A1 gene expression (mRNA levels) in Caco2 cells by isomeric flavones and isoflavones with the same substitution patterns.

A list of natural products containing flavonoids that improve cancer immunotherapy, including cancer vaccines, immune-check points inhibitors, and adoptive cell transfer therapy is shown in Table 4.

**Table 4:** List of natural products containing flavonoids that improve cancer immunotherapy including cancer vaccines, immune-checkpoints inhibitors, and adoptive cell transfer therapy PD-L1: Programmed death ligand 1; JAK/STAT: Janus kinase/signal transducers and activators of transcription

Source	Natural product	Key Points of Sensitizing Immunotherapy	References
Hedyotis diffusa	Rutin	Enhance the anticancer effect of cancer peptide-based vaccines	[134]
Citrus maxima	Naringenin	Enhance the anticancer effect of cancer vaccines	[135]
Silybum marianum	Gaertn. Silibinin	Downregulate the expression of PD-L1 through JAK/STAT pathway	[136]
Scutellaria baicalensis	Baicalein Baicalin	Downregulate the expression of PD-L1 through JAK/STAT pathway	[137]

## Conclusions

Whereas stromal fibroblasts have been shown to effectively limit metastasis in individuals with cancer, other mechanisms of niche creation are likely to exist and must be studied. Although there has been a lot of promising research on the subject of TME, translation of these findings to the clinic will require more work before they can be used. We need to learn more about the association of CAFs with pre-metastatic niche and co-culture experiments with stromal components (fibroblast, ECM, or immunologically positive myeloid cells). Many sophisticated preclinical research now use cancer epithelia targeting techniques. The development of target therapies for CAF, on the other hand, could be worthwhile. The initial hurdle would be defining the CAF population and distinguishing it from wound healing and inflammatory activities that naturally occur. The metastatic niche should be a focus for therapeutic targets to prevent colonization and proliferation at the secondary site. This area of targeting may be used to supplement present and future treatment techniques that target tumor cells reducing side effects, medication resistance, and recurrence.

From the review of the articles, we conclude that TQ has anticancer activity through modulation of the tumor environment by inhibiting CAFs. We also conclude that other flavonoids have anticancer properties, especially through tyrosine kinase inhibition.
